# Atypical Presentation of Cerebral Palsy and Seizures: A Case Report on Rasmussen’s Encephalitis in an Adolescent

**DOI:** 10.7759/cureus.13705

**Published:** 2021-03-04

**Authors:** Naveed S Noordin, Logan J Deyo, Connor W Ryon, Willie T Anderson

**Affiliations:** 1 Department of Pediatrics, East Tennessee State University James H. Quillen College of Medicine, Johnson City, USA

**Keywords:** rasmussen encephalitis, pediatric seizure, atypical presentation, pediatric rare diseases

## Abstract

Rasmussen’s encephalitis is a rare neurological disease first described in 1958 that is characterized by medico-refractory seizures, focal unilateral cerebral inflammation, and deficits such as hemiparesis. While we still do not have a full understanding of this disease, proposed theories behind its etiology include auto-immune manifestations, immune attack by T cells, and malfunctional alterations in genetic expression. It is classically considered a rare childhood malady with a median age of onset of six years, and cases in adolescents and adults are even rarer, representing up to 10% of all cases to date. In this report, we would like to share a rare case of Rasmussen's encephalitis that occurred in an adolescent. Our 17-year-old male patient presented with signs and symptoms beginning at age 14 and was initially diagnosed with cerebral palsy only to later present with additional symptoms and characteristic EEG and MRI findings that ultimately led to a diagnosis of Rasmussen’s encephalitis. Thus, with this case report, our intent is twofold: to shed light on an atypical presentation of an already rare disease, even rarer in adolescents and adults, and to underscore the importance of keeping a broad differential when it comes to evaluating a patient with seizures.

## Introduction

In their pioneering paper from 1958, Rasmussen et al. described cases of a neurological malady consisting of focal seizures, unilateral cerebral hemispheric inflammation, and subsequent impairment including hemiparesis. They presented the first case of what we now understand as Rasmussen’s encephalitis. While the pathobiology of this disease process is still incompletely understood, some proposed hypotheses to explain its etiology include T-cell mediated cytotoxicity, antibody-mediated central nervous system (CNS) inflammation, and cellular anomalies related to aberrant gene expression [1-11]. Classically considered a childhood disease with a median age of onset of six years, it consists of three stages in the presentation: a prodromal phase that could last a few years and includes isolated seizures and mild hemiplegia, an acute phase that follows with simple partial seizures and often epilepsa partialis continua (EPC) mainly arising from a single hemisphere, and lastly a residual phase with often pharmaco-refractory epilepsy as well as permanent motor and cognitive deficits [4]. EEG and MRI are often helpful in diagnosis along with the clinical presentation. EEG in patients with Rasmussen’s encephalitis often shows abnormal slow-wave discharges and abnormal delta activity with irregular spike and wave phenomenon, which occur in concert with irregular epileptiform discharges that occur in the interictal period. MRI often shows marked atrophy as well as increased signal intensity from the affected unilateral hemisphere [5]. Treatment of this neurological malady often involves a combination of immunotherapy, antiepileptic medications, and then hemispherectomy in cases of treatment-resistant epilepsy [2,7,11].

As mentioned, Rasmussen's encephalitis classically presents and is diagnosed in childhood, and a new presentation and diagnosis of Rasmussen's encephalitis is much less common in adolescents and adults, and only about 10% of all cases of Rasmussen's encephalitis have been documented in these latter groups, with the oldest reported diagnosed patient being 58 years [1,6,10]. And while rare in adolescents and adults, it is associated with a better overall clinical course and prognosis with less severe cognitive deficits and hemiparesis but with a higher association with temporal and occipital lobe epilepsy [9,10]. In this case report, we would like to present a case of a 17-year-old male who presented with a clinical syndrome and brain imaging consistent with a diagnosis of Rasmussen’s encephalitis.

## Case presentation

A 17-year-old male presented to the emergency department (ED) due to new onset seizure activity. The patient had some prior neurological concerns which began with difficulty achieving major developmental milestones. He did not learn to walk until 17 months of age and required speech therapy at the age of two. Whether these details were relevant to his future diagnosis of Rasmussen’s encephalitis is unclear but it does show that he had neurological impairment from an early age, and additionally the patient had a maternal aunt with a past medical history of seizures. At age 11, he first noticed signs of right hemilateral weakness beginning in the upper extremity. By age 14, his neurological symptoms continued to progress leading to complete right hemilateral weakness causing him to hold his arm in a “drawn-up” fashion and walking with a limp, prompting the need to obtain an MRI at age 14. This MRI was read as a cerebral vascular accident and the patient was told that he would probably have residual hemilateral weakness and was diagnosed with cerebral palsy.

A week before our patient arrived at the ED he had his first seizure around 0230 in the morning. Our patient was in the kitchen and found by his parents complaining that he could no longer feel or move his right upper and lower extremities. He did not lose consciousness and had no post-ictal state. Neither the patient nor his parents thought much of it so he returned to bed and woke up feeling normal the following morning. On the day of admission, our patient had a second simple partial seizure around 0730 while brushing his teeth. The patient stated he had aura and presyncope. He sat down and called for his parents who noted he had rhythmic jerking movements of both the right upper and lower extremities lasting 4-5 minutes. Our patient did not lose consciousness or bowel/bladder function and was able to maintain brief conversations during the event and recall the details after although he did undergo a period of Todd’s paralysis.

**Figure 1:**
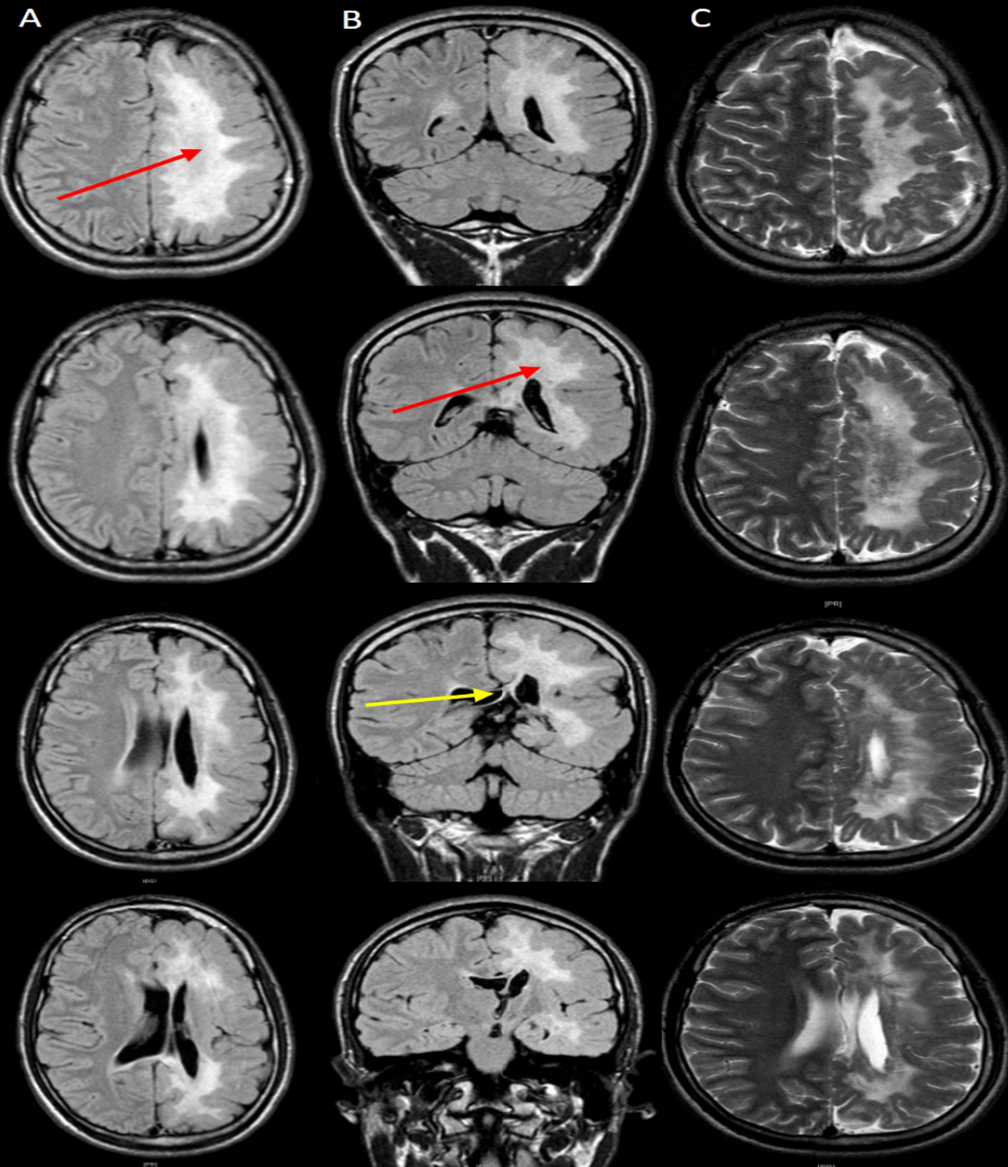
T1 and T2-weighted MRIs in columns A, B, and C, respectively, show left cerebral hemisphere diffuse white matter loss (red arrows) and midline shift (yellow arrow) due to corpus callosum volume loss.

In the ED, our patient reported continued numbness and twitching in his right upper extremity that had persisted for 3-4 hours after the seizure. Our patient’s physical exam findings were pertinent for 3+ reflexes on the right side and 4/5 strength. All other physical exam findings were within normal limits. Following admission, our patient was started on oxcarbazepine and also underwent MRI with and without contrast that showed diffuse white matter disease involving the entire left supratentorial cerebral hemisphere with associated predominant white matter volume loss and severe diffuse volume loss involving the corpus callosum (Figures 1A-1C). These changes were highly suggestive of Rasmussen’s encephalitis and had also caused lateral ventricle asymmetry leading to a leftward 8mm interventricular septum deviation due to diffuse volume loss.

Our patient and family were informed of the results and instructed to follow-up with another children’s hospital capable of providing a wider variety of treatment options including hemispherectomy as Rasmussen’s encephalitis is a progressive disorder that is frequently refractory to medical therapy alone [2,7,11].

## Discussion

With our case we highlight an atypical presentation of late-onset cerebral palsy and seizures, leading ultimately to a diagnosis of Rasmussen’s encephalitis in an adolescent. This disease primarily arises and is diagnosed in childhood and is characterized by focal epilepsy, worsening neurological functioning, and eventual hemiparesis and severe encephalopathy [9]. The literature reports that 10% of Rasmussen’s cases are accounted for by adolescents and adults with a milder, more protracted clinical course [1,6,10]. EEG and MRI are diagnostic in patients with Rasmussen’s encephalitis often showing abnormal slow-wave discharges and delta activity with irregular spike and wave phenomenon, and extensive inflammation and white matter atrophy with ventricular enlargement consolidated to a single hemisphere of the brain [5]. Rasmussen’s encephalitis is unique as these symptoms tend to be refractory to typically highly effective anti-epileptic medications, and an effective treatment option is usually surgical intervention via hemispherectomy, which has its own associated benefits, risks, and contraindications [2,7,11]. A study done following the clinical course of 10 atypical cases of Rasmussen’s encephalitis showed hemispherectomy being effective in controlling seizures and motor and mental deterioration in over 80% of patients with partial recovery of neurological deficits [2,3,7,11].

Our patient presented to the ED reporting two focal seizures at home: one seizure a week prior to admission with accompanied brief loss of sensation and paralysis to the right upper and lower extremities and another the morning of admission with rhythmic jerking on the right side. On obtaining a further history, we learned that our patient was late in meeting developmental milestones, including language and gross motor skills, and that he began having complaints of unilateral weakness through his teenage years and was subsequently diagnosed with cerebral palsy upon workup when he was 14 years old. Physical exam showed increased reflexes and decreased strength in the right upper and lower extremities. EEG findings displayed characteristic left hemisphere delta slowing with irregular spike and wave phenomenon. MRI was highly suggestive of Rasmussen’s encephalitis with findings of extensive white matter loss involving the entire left supratentorial cerebral hemisphere and interventricular septum deviation. Our patient was referred to an out-of-state children’s hospital with the additional services needed for further evaluation and treatment, including the option of hemispherectomy.

## Conclusions

Although our patient’s case is still in progress and treatment is ongoing, our patient’s symptom onset and initial presentation as well as his clinical progression are nevertheless atypical and unique. Due to its rarity, adolescent-onset Rasmussen’s encephalitis is vastly underrepresented in the literature and thus, an afterthought as a differential diagnosis for late-onset cerebral palsy and seizures. Our goal is to shed light on a puzzling case and call for the medical community to be cognizant of rare possibilities within their differentials.
